# Stopping at a red light: Recruitment of inhibitory control by environmental cues

**DOI:** 10.1371/journal.pone.0196199

**Published:** 2018-05-03

**Authors:** Shachar Hochman, Avishai Henik, Eyal Kalanthroff

**Affiliations:** 1 Department of Psychology, Ben-Gurion University of the Negev, Beer-Sheva, Israel; 2 Zlotowski Center for Neuroscience, Ben-Gurion University of the Negev, Beer-Sheva, Israel; 3 Department of Psychology, The Hebrew University of Jerusalem, Jerusalem, Israel; Eberhard-Karls-Universitat Tubingen Medizinische Fakultat, GERMANY

## Abstract

Environmental cues can influence basic perceptual and attentional processes especially in an emotional context. In the current study, we aimed to investigate the effect of a non-emotional common environmental cue―a traffic light—on a higher cognitive operation―inhibition. In two experiments, we administered a novel version of the stop-signal task, in which the go task was to determine the color of a traffic light. In order to investigate the influence of each of the cues on inhibitory processes, separate tracking procedures (one for each cue) were applied simultaneously to the stop-signal delay. In Experiment 1, we found that reaction time in no-stop-signal trials was faster when a green traffic light was present, whereas stop-signal reaction time was longer when a red traffic light was present. In Experiment 2, neutral control cues were used in addition to a red and green light. The results indicate that the differences between red and green stem from an association between the color red and stop processes (rather than from the green-go association). These results strengthen previous findings showing the effect of environmental cues on attentional processes and go beyond them by showing that the effect is not restricted to emotional cues. Most importantly, the current study results suggest that environmental cues can also influence complex cognitive operations such as inhibitory control. These results might have specific implications for our understanding of the processes that underlie specific psychiatric disorders characterized by inhibitory deficit.

## Introduction

Environmental cues trigger a set of behaviors that are essential for adaptive behavior. For example, when driving a car, the color of the traffic light serves as a cue that signals whether we should stop the car or continue/start driving. These actions (i.e., braking or continue driving) are voluntary and conscious, and can be controlled or changed according to the situation (e.g., going through a red light when a right turn is permitted). Environmental cues can also trigger automatic, involuntary and unconscious processes that can influence behavior. For example, presenting alcohol-related stimuli to alcoholics triggers a set of involuntary behaviors specifically related to these stimuli [1]. In fact, it has been shown that many stimuli can trigger motor codes of behaviors that are strongly associated with these stimuli, possibly even unconsciously [2, 3, 4]. Despite the vast amount of research showing that environmental cues influence basic attentional processes and action tendencies, only a few studies investigated whether these cues recruit high cognitive operations, such as inhibitory control, which are crucial for adaptive behavior.

Evidence for the automatic influence of environmental cues on attention and behavior comes from several lines of research. A vast amount of studies have previously shown that environmental cues (e.g., eyes gaze, arrows, threatening stimuli) cause reflexive orienting of attention [5, 6, 7], modulate visual search efficiency [8], and influence approach and avoidance behaviors [9]. A well-documented effect of environmental cues on behavior comes from priming, and especially affective priming, tasks. In these tasks, presentation of a prime that triggers a specific attitude results in facilitated reaction time (RT) for a following target that triggers the same attitude [10, 11]. Thus, it is believed that environmental cues automatically activate specific attitudes or goals. Environmental cues can also influence motor planning and action execution. For example, individuals with alcohol dependency have difficulties when they are requested to move a picture of alcohol away by pushing a joystick [12]. However, little is known about the ability of environmental cues to trigger executive processes, such as inhibitory control.

Inhibitory control is considered to be a high-order cognitive operation that enables goal-directed behavior by suppressing or stopping irrelevant information or behavior. One common task to measure inhibitory control in the lab is the stop-signal task [13]. In this task, participants are asked to rapidly respond to visual stimuli (go signals) knowing that on some trials an auditory stimulus (stop signal) will be presented, requiring them to inhibit their already-initiated (prepotent) response. This paradigm estimates the ability to inhibit prepotent responses by calculating participants’ stop-signal reaction times (SSRTs)—an index of inhibitory control [13]. Longer SSRTs have been associated with deficient response inhibition. According to the ‘horse race model’, suggested by Logan, Cowan and Davis [14, 15], the two processes—go and stop—compete with each other and thus they are generally independent. The SSRT represents the stop process while the nsRT (no-stop RT) represents the go process [16, 17].

**Fig 1:**
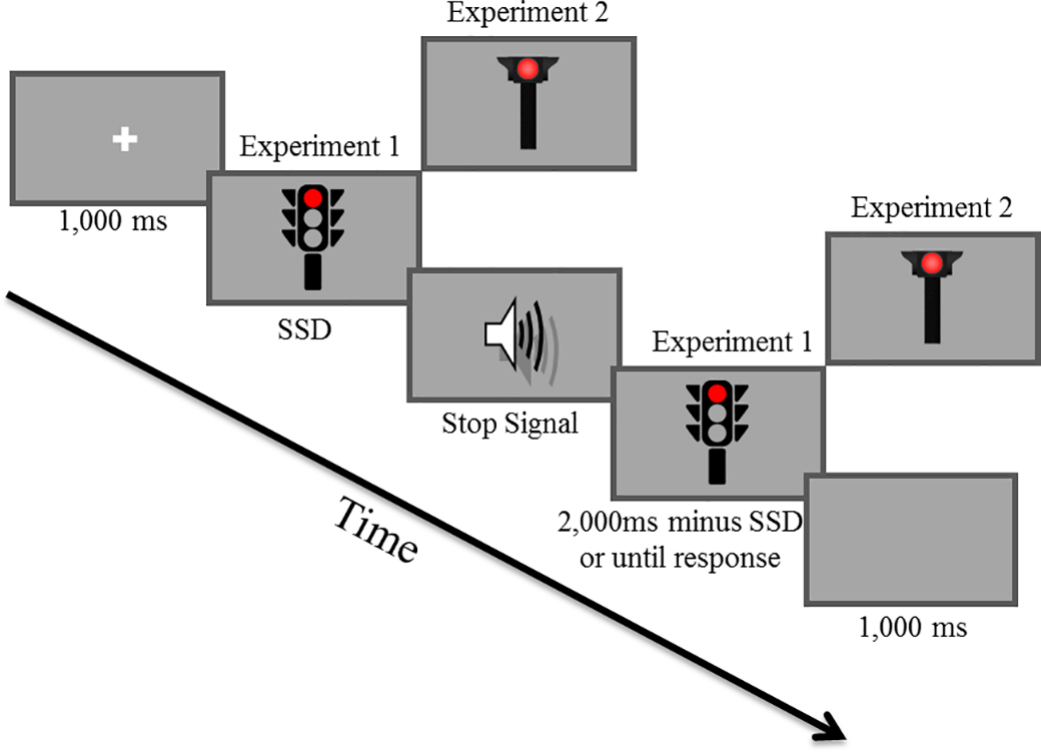
Example of a red traffic light stop-signal trial. In both experiments, each trial started with a 1,000 ms fixation (a white plus sign at the center of a gray screen). Following this, a visual go stimulus appeared (i.e., picture of red, green, or black (the latter only in Experiment 2) traffic light that appeared randomly and in equal proportions). In no-stop-signal trials, the go stimulus stayed in view for 2,000 ms or until a key press. In stop-signal trials, an auditory tone was presented shortly after the appearance of the go-signal. The duration between the go- and stop-signal (SSD; stop-signal delay) was subjected to a tracking procedure that was applied separately for each type of stimulus (red or green traffic light). Each trial ended with a 1,000 ms inter-trial interval (gray screen). Examples of a red light stimuli are presented from Experiment 1 (3-aspects traffic light) and Experiment 2 (1-aspect traffic light).

In the current paper, we aimed to investigate the effect of an environmental cue (picture of a red or a green traffic light) on inhibitory control. Verbruggen and Logan (Experiment 1 & Experiment 2) [18] tested the effect of task-irrelevant cues—the written words STOP or GO presented inside the go-signal (i.e., white square or circle)—on performance in the stop-signal task. While these researchers found slower nsRTs for the STOP cue compared to the GO cue, they found no differences between the two conditions for SSRTs. The authors suggested that the latter might be due to the fact that the word-cue was task-irrelevant (and was not required to be processed). Thus, they conducted another experiment in which the word-cue was presented as the stop signal. In this experiment, the authors found that SSRT was longer for the GO cue than for a neutral cue but there was no difference between the SSRT of the neutral cue and the STOP cue, indicating no automatic inhibition triggered by the STOP cue. These surprising results might be due to three reasons: (a) the content of the word-cue was always irrelevant and thus, was still not required to be processed—participants were instructed to inhibit the go response whenever any of these stimuli appeared, (b) the word-cue was associated with the stop signal instead of the go signal, leaving significantly less time for priming processes to influence response, and most importantly, (c) the word-cue was a written word that might not have been associated strongly enough with stopping. We believe that word cues have significantly smaller effect compared to environmental cues. This idea relies on the suggestion that the stimulus might trigger tasks that have acquired a strong association with it [3, 19, 20, 21] and that the task that is most associated with words is reading [22, 23]. The current study used a modification of the stop-signal task in order to further examine the influence of environmental cues on action potentiation and response inhibition in the stop-signal task (see Fig 1). With that aim in mind, in the current study the cue was the go stimulus and thus task relevant (i.e., required maximum processing). In addition, we used colored traffic lights, which are strongly associated with stopping and going and require almost no preliminary processing (i.e., reading) to trigger the stop or go tasks. Importantly, although stopping a car involves a set of behaviors (e.g., stopping to press the gas pedal and pressing the brake pedal), the association between a red traffic light and the concept of stopping is based on the fact that ‘stopping at a red light’ is a notion that is relevant to all people using streets and roads.

Based on previous findings showing that environmental cues modulate simple and complex action-execution processes and executive functions, we predicted that nsRTs to a green traffic light would be shorter than RTs to a red traffic light. Most importantly, we predicted that in the current design when a red traffic light appears, stopping will be easier and quicker than stopping when a green traffic light appears (i.e., shorter SSRT in red traffic light trials compared to green traffic light trials). This result would indicate that environmental cues modulate complex inhibitory control when they are strongly associated with stopping and are properly processed.

## Experiment 1

In this experiment, we used a traffic light with two colors—red and green—as go signals in a modulated stop-signal task (see Fig 1). Notably, the stimuli were task-relevant and content-relevant.

### Materials and methods

#### Participants

Twenty students (13 females and 7 males) of Ben-Gurion University of the Negev (Israel) participated for a small monetary payment (about 10$). The study was approved by the ethics committee of the Psychology department of Ben-Gurion University of the Negev (Israel) and all participants signed an informed consent form prior to their participation in the experiment. All participants had normal or corrected-to-normal vision, had no reported history of attention deficit/hyperactivity disorder or learning disabilities, and all were naive as to the purpose of the experiment. The youngest participant was 19 years old and the oldest was 27 years old (mean = 23.75 years, standard deviation (*SD*) = 2.36, with no difference between genders).

A power analysis using G-Power 3.1 [24], based on the effect sizes reported in previous studies (in [18], Experiment 1, $\eta_{p}^{2}$ = .18), indicated that the current sample (*N* = 20) allowed for examination of group differences in SSRT and nsRT at a power > 80% to test small-to-medium effect sizes with a Type I error (α < .05).

#### Stimuli

The go signal was a picture of either a red or a green traffic light (see Fig 1). The pictures were presented at the center of a screen on a gray background and were 11.5'' high and 6.8'' wide. The stop signal was an auditory tone (750 Hz, 75 ms) delivered by headphones.

#### Procedure

Data collection and stimulus presentation were controlled by a DELL OptiPlex 760 vPro computer with an Intel core 2 duo processor E8400 3 GHz. Stimuli were presented on a DELL E198PF 19″ LCD monitor. A keyboard was placed on a table between the participant and the monitor. Participants were tested individually and sat approximately 40'' from the computer screen. Red and green colored stickers were taped on the 'q' and 'p' keys (counterbalanced), which served as response keys. The experiment included 40 practice trials (10 of which were stop trials), which included feedback for speed and accuracy and were not further analyzed, and 480 experimental trials (120 of which were stop trials). Participants were told that the practice block would be identical to the experimental block but that the experimental block would be longer and would not include feedback (see Fig 1 for further details). An auditory stop signal appeared in a random selection of 25% of the trials and red or green lights appeared in equal proportions. The stop signal was presented after a variable stop-signal delay (SSD) that was initially set at 250 ms and was continuously adjusted according to separate staircase tracking procedures that were applied for each type of stimulus (red or green traffic light) to obtain a probability of stopping of 50% for the green and red light separately; after each successful stopping (following a stop signal) the SSD was extended by 20 ms and after each unsuccessful stopping the SSD was shortened by 20 ms. Participants were asked to do a manual two-color (red vs. green) discrimination task and the instruction indicated to press the left/right key with the corresponding index finger, as quickly and accurately as possible, and emphasized not to wait for a potential stop signal. RT was calculated from the appearance of the go stimulus to the response. Trial order was randomized with two restrictions: we had the same number of red and green traffic light stop-signal trials (60 of each), and we had the same number of red and green traffic light no-stop-signal trials that followed stop-signal trials (60 of each; Fig 1).

### Results

In order to investigate our first hypothesis that responding to a green traffic light would be faster than responding to red traffic light, mean RTs of correct responses were calculated for no-stop-signal trials for each participant in each condition (red vs. green traffic light). A one-way analysis of variance (ANOVA) with repeated measures was applied to nsRT data (i.e., RT for no-stop trials) with condition as a within-subject factor. The nsRT for a green traffic light was shorter than the nsRT for a red traffic light, *F*_(1, 19)_ = 12.53, *MSE* = 568.88, *p* < .01, $\eta_{p}^{2}$ = .40 (see Table 1). A similar one-way ANOVA for accuracy rates in no-stop-signal trials after arcsine-transformation revealed no significant difference between green and red traffic lights, *F*_(1, 19)_ = 1.79, *MSE* < 0.1, *p* = .20, $\eta_{p}^{2}$ = .08.

**Table 1:** Results of the different traffic light conditions in no-stop-signal and stop-signal trials.

		Traffic Light Conditions				
		Experiment 1			Experiment 2	
Trial Type		Green	Red	Green	Black	Red
No-Stop						
	nsRT	468 (21)	494 (20)	595 (13)	593 (12)	606 (12)
	ACC	.98 (< .01)	.98 (< .01)	.95 (< .01)	.97 (< .01)	.95 (< .01)
Stop-Signal						
	SSRT	183 (16)	164 (17)	256 (10)	258 (10)	241 (10)
	p(r|s)	.48	.46	.47	.46	.46
	SSD	254 (23)	293 (23)	311 (14)	310 (14)	334 (15)
	srRT	133 (16)	128 (16)	199 (9)	192 (9)	189 (9)

*Note*. *R*eaction time in milliseconds (one standard error of the mean); nsRT = reaction time of correct responses for no-stop trials; ACC = accuracy rates; SSRT = stop-signal reaction time; p(r|s) = proportion of erroneous responses to stop-signal trials; SSD = stop-signal delay (duration between the go and stop signals); srRT = reaction time for erroneous response to stop-signal trials.

To examine our second hypothesis that stopping for a red light would be faster than stopping for a green light, SSRT was calculated using the integration method [17, 25]. No-stop-signal trial RTs were determined by the *n*^th^ RT, that is, N (number of correct no-stop-signal trials) × p(response|signal), which was calculated for each participant in each condition separately. SSRT was then calculated as the *n*^th^ RT-mean SSD (which was adjusted for each participant in each condition separately). SSRT data were subjected to a one-way repeated-measures ANOVA with condition (red vs. green traffic light) as a within-subject factor. SSRT for a red traffic light was found to be significantly shorter than SSRT for a green traffic light, *F*_(1, 19)_ = 6.96, *MSE* = 513.37, *p* < .02, $\eta_{p}^{2}$ = .27 (see Table 1), indicating that inhibiting a response was faster when a red traffic light appeared.

Finally, RT for erroneous responses to stop-signal trials (i.e., signal-respond RT; srRT) was shorter than nsRT for all subjects, confirming that the independence assumption of the race model was not violated (see Table 1; for a recent discussion of this issue, see Verbruggen & Logan [26]).

## Experiment 2

Experiment 1 demonstrated the effect of environmental cues both on go process (nsRT differences) and on stop process (SSRT differences). Yet, it is unclear whether the differences found between green and red result from automatic inhibition due to a red-stop association or from an automatic facilitation due to a green-go association. Hence, we employed a similar task with a control neutral cue—a black light. We refrained from using yellow as neutral as in real life, this color in a traffic light is associated with a specific behaviors (i.e., preparing to stop), hence would not be appropriate neutral. In addition, in Experiment 2 we used a 1-aspect traffic light given that a 3-aspects traffic light would have forced us to place the black color in the position of the central light giving it an irrelevant advantage.

### Materials and methods

#### Participants

Thirty-two students (19 females and 13 males) from Ben-Gurion University of the Negev (Israel) participated for a small monetary payment (about 10$). The study was approved by the ethics committee of the Psychology department of Ben-Gurion University of the Negev and all participants signed an informed consent form prior to their participation in the experiment. Exclusion/inclusion criteria were identical to those in Experiment 1. Two participants (1 female and 1 male) were excluded due to high standard deviations and abnormal performances. The youngest participant in the experiment was 21 years old and the oldest was 28 years old (mean = 24.70 years, *SD* = 1.95, with no difference between genders).

A power analysis using G-Power 3.1 [24], based on the SSRT effect size reported in Experiment 1, indicated that the current sample (*N* = 30) allowed for examination of group differences at a power > 81% to test small-to-medium effect sizes with a Type I error (α < .05).

#### Stimuli

The go signal was a picture of either a red, black or green light within a single aspect traffic light (see Fig 1). Except for this, all the stimuli in Experiment 2 were identical to those in Experiment 1.

#### Procedure

In Experiment 2, participants had to carry out a manual three-color discrimination task (red vs. black vs. green). Red, black and green colored stickers were taped on the 'f', 'g' and 'h' response keys of a computer keyboard. The black color was always on the 'g' key whilst red and green keys changed between participants (counterbalanced). This experiment included 48 practice trials (12 of which were stop trials), which included feedback for speed and accuracy and were not further analyzed, and 720 experimental trials (180 of which were stop trials). Except for this, all procedures in Experiment 2 were identical to those in Experiment 1. Separate staircase tracking procedures were applied for the three colors.

### Results

In order to investigate the go processes triggered by the three traffic lights, a one-way ANOVA with repeated measures was applied to nsRT data with condition (red vs. black vs. green traffic light) as a within-subject factor. There was a significant difference between the three conditions, *F*_(2, 58)_ = 3.53, *MSE* = 407.4, *p* < .04, $\eta_{p}^{2}$ = .11 (see Table 1). In addition, planned comparisons revealed that nsRT for the red traffic light was significantly slower than for the black traffic light, *F*_(1, 29)_ = 8.62, *MSE* = 288.3, *p* < .01, $\eta_{p}^{2}$ = .22, while there was no significant difference between nsRT for the green and black traffic light, *F* < 1. Importantly, the current results replicated the findings from Experiment 1, in which response to a red traffic light was significantly slower than to a green traffic light, *F*_(1, 29)_ = 4.24, *MSE* = 417.1, *p* < .05, $\eta_{p}^{2}$ = .13. A similar one-way ANOVA for accuracy rates in no-stop-signal trials after arcsine-transformation revealed no significant effect for condition, *F*_(2, 58)_ = 2.73, *MSE* < 0.01, *p* = .07, $\eta_{p}^{2}$ = .08.

As in Experiment 1, SSRT was calculated using the integration method (see description above). SSRT data were subjected to a one-way repeated-measures ANOVA with condition (red vs. black vs. green traffic light) as a within-subject factor. There was a significant difference between the conditions, *F*_(2, 58)_ = 3.53, *MSE* = 698.3, *p* < .04, $\eta_{p}^{2}$ = .11. Planned contrasts to test our a priori hypotheses revealed shorter SSRT for a red traffic light compared to a black traffic light, *F*_(1, 29)_ = 4.80, *MSE* = 826, *p* < .04, $\eta_{p}^{2}$ = .14, but no significant differences between a green traffic light and a black traffic light, *F* < 1 (see Table 1). Similarly to the results in the nsRT, the current results replicated the findings from Experiment 1, in which response to a red traffic light was shorter than to a green traffic light, *F*_(1, 29)_ = 5.17, *MSE* = 659.7, *p* < .04, $\eta_{p}^{2}$ = .15.

Finally, RT for erroneous responses to stop-signal trials (i.e., signal-respond RT; srRT) was shorter than nsRT for all subjects, confirming that the independence assumption of the race model was not violated (see Table 1; for a recent discussion of this issue, see Verbruggen & Logan [26]).

## Discussion

In the current study, we aimed to investigate whether contextual environmental cues (i.e., a traffic light) could modulate simple action execution processes as well as complex cognitive operations, such as inhibition of a prepotent response. In two experiments, we used a modified stop-signal task, in which two (Experiment 1) or three (Experiment 2) separate tracking procedures were applied. In Experiment 1, we found that RTs for a green traffic light were shorter than RTs for a red traffic light. Most importantly, we found that stopping was reliably different when a red traffic light was presented compared to when a green traffic light was presented. Experiment 2 revealed that both these effects were mainly driven from the red traffic light. In this experiment, we found that RTs for a red traffic light were slower than RTs for a black (neutral) traffic light, whilst there was no difference in RTs between the black and green traffic light. Similarly, we found that SSRTs for the red traffic light were faster than SSRTs for the black traffic light, whilst there was no difference in SSRTS between the black and green traffic light. Notably, the differences in response between the green and red traffic lights from Experiment 1 were replicated in Experiment 2.

The first finding—shorter RTs to a green traffic light—reinforces conclusions from previous studies that found environmental cues can influence basic cognitive and attention processes [e.g., 5, 6 (Experiment 3a), 7, 8]. It has been shown that emotional stimuli can guide attentional and perceptual processes by increasing their priority and thus causing enhancement of visual processing at affectively cued locations [27, 28, 29]. The current study provides novel evidence that a neutral, every-day stimulus, such as a traffic light, can have a similar effect on higher, more executive aspects of the attention system. Specifically, our results indicate that a red traffic light might increase the priority of a specific process (stop) and thus prime or enhance this process. This strengthens the suggestion that environmental cues affect higher attention processes, and that this effect is not necessarily unique to affective cues.

The most important finding of the current study, underlined in Experiment 2, is that it is easier (and faster) to stop when a stopping-related environmental cue—a red traffic light—appears compared to when a going-related environmental cue—a green traffic light—appears or when a neutral cue appears—a black traffic light. This indicates that environmental cues can influence complex cognitive operations, such as inhibition of a prepotent response. Our data suggest that the ‘horse race model’ assumption regarding the independency of go and stop processes [14, 15] was not violated in either experiment. Though it might be intuitive to think that SSRT would be shorter in conditions that require long RTs (one has more time to stop, hence it is easier), it has been shown that more complicated (or emotional) tasks allowed less free resources so that SSRT was prolonged even though RTs in these conditions were longer [30, 31, 32, 33]. In the current study, all tasks (responding to a green, red or a black traffic light) were equally complicated, thus our results suggest that not only longer SSRTs cannot be attributed to shorter RTs, but also that they cannot be attributed to difference in task complexity.

Shorter SSRT when a red traffic light is presented can be discussed in terms of priming or increasing the priority of the stopping process. Notably, it has been suggested that ‘automatic inhibition’ (i.e., the re-instantiation of response inhibition via retrieval of stimulus-stop associations [34]) can be acquired via learning and conditioning [35, 36]. For example, it has been demonstrated that images of food and alcohol can become associated with stopping after a training phase [37, 38]. The only study that addressed this association using neutral, not learned, stimuli was by Verbruggen and Logan [18]. These researchers found that the task-irrelevant word-cues STOP and GO affected RTs on no-stop-signal trials but not SSRT. In the current study, we showed that using *task-relevant* everyday environmental stimuli might yield different results—an effect on both nsRT and SSRT (i.e., going and stopping processes). A red-traffic light is likely to associatively evoke a strong stopping task, which in turn primes the stopping process in the stop-signal task. More generally, the current study results imply that inhibition can also be automatically evoked in the face of a cue that is strongly associated with stopping. In this case, inhibition was primed by the red traffic light and hence, this made it easier to stop a prepotent response that was irrelevant to the traffic light.

Beyond elaboration of the knowledge regarding the influence of environmental cues on going and stopping behaviors, the current study results might have implications on our understanding of various psychopathologies. Failure to inhibit certain behaviors might result in execution of unwanted behaviors or difficulties to stop irrelevant behaviors. Thus, deficient inhibitory control has been suggested to be a core factor in several psychopathologies [39, 40, 41, 42, 43] that are often conceived to be ‘behavioral addictions’ [44]. These include substance abuse [45, 46, 47], obsessive-compulsive disorder (OCD), trichotillomania [48, 49, 50], eating disorders [51], and gambling [46]. Importantly, the tasks that are used to examine inhibition impairment commonly consist of meaningless cues, whereas the symptoms of these disorders are commonly linked to specific environmental cues. These cues can include, for example, bottles of alcohol (for individuals suffering from alcohol abuse), cakes (binge eating), or cleaning materials (OCD). Some of these environmental cues might facilitate inhibitory processes (similar to the red traffic light in our study). For example, while cleaning materials may trigger a go-behavior (hand washing) for an OCD patient, a public bathroom’s door handle may trigger inhibition (do not reach) in the same patient. Similarly, while a cake may trigger a go-behavior (eating) for a bulimic patient, it might trigger inhibition for an anorectic patient (do not eat). Better understanding of these automatic processes might lead to new treatment targets for these patients [52]. Importantly, considering that the current study did not test psychopathological patients, these suggestions should be considered with caution.

The current study results indicate that environmental cues affect inhibitory processes. Specifically, our results indicate that some environmental cues might trigger automatic inhibitory process. This might shed new light on the inhibitory processes that underlie several disorders characterized by deficient inhibition. Future study should investigate the effect of ‘disorder-specific’ stimuli on the ability of individuals to inhibit responses.
